# Enhanced Flame-Retardant Properties of PVDF Using a Multiphase Synergistic Approach with Phytate-Chitosan-Modified Boron Nitride

**DOI:** 10.3390/polym17212904

**Published:** 2025-10-30

**Authors:** Shiyi Ming, Piao Wang, Shaoyuan Wu, Jinghan Hu, Jie Zhang, Lianlian Li, Bingyue Huang, Weijiang Huang, Xingyu Guan, Kui Wang, Wei Yan

**Affiliations:** School of Materials Science and Engineering, Guiyang University, Guiyang 550005, China; 17311863003@163.com (S.M.); 15286500235@163.com (P.W.); 18785593078@163.com (S.W.); pipihanhu@163.com (J.H.); z2601945295@163.com (J.Z.); 15721759465@163.com (L.L.); 18984355256@139.com (B.H.); huangweijiang_2007@126.com (W.H.); gxy712@163.com (X.G.); gyxywkui@163.com (K.W.)

**Keywords:** phytic acid, chitosan, boron nitride, polyvinylidene fluoride, flame retardancy

## Abstract

The morphology and composition of inorganic particles play a vital role in controlling the flame-retardant characteristics of polymers. Halogen-free flame-retardant polymers have also become a current research hotspot. Boron nitride (BN), phytic acid (PA), and chitosan (CS), a natural polysaccharide with a nitrogen content of approximately 6.8–7.5%, show great promise as flame retardants owing to their high thermal stability, P-based flame retardancy, and natural polysaccharide properties, respectively. In this study, BN (BN@PA-CS) particles coated with PA and CS were designed and prepared via a facile modification strategy. The effect of BN@PA-CS on the mechanical and flame-retardant properties of polyvinylidene fluoride (PVDF) was further investigated, and it was found that both characteristics were improved. Compared to pure PVDF, the PVDF composite films exhibited a significantly lower peak heat release rate and total heat release. With a BN@PA-CS content of 20%, the peak was the lowest at 18.25 W/g, corresponding to a decrease of 77.83%. This phenomenon may be attributed to the synergistic effect of the BN nanosheets and PA-CS in the BN@PA-CS particles. This work describes a facile and effective method of modifying the morphology and composition of inorganic particles, thereby controlling the properties of polymers, and provides a new approach to improving the safety of PVDF battery separators.

## 1. Introduction

Polyvinylidene fluoride (PVDF) is a high-performance fluorinated polymer material that exhibits a range of excellent properties. Its high chemical resistance, superior electrical insulation, good mechanical strength, and unique piezoelectric/pyroelectric characteristics have made it indispensable in critical applications across fields such as energy, electronics, environmental protection, and chemical [1,2,3]. Particularly in the rapidly evolving energy sector, electric vehicles are becoming increasingly popular and have been widely adopted in recent years owing to their clean and efficient characteristics. As the core power source for new energy vehicles, lithium-ion batteries have played a pivotal role [4,5]. PVDF, with its excellent electrolyte affinity, has been widely used as battery separator membranes. However, lithium-ion batteries in new energy vehicles generate high temperatures during operation, causing the battery separator membranes to contract, which has led to safety concerns [6,7,8,9].

To enhance the safety of lithium-ion batteries, many studies have focused on PVDF battery separator membranes [10,11,12,13,14,15,16]. For instance, modifications such as introducing metal–organic framework materials into PVDF have been used to reduce interfacial resistance and minimize heat generation [17,18,19]. In other studies, silica (SiO_2_) and polyetherimide (PEI) were combined with PVDF to form composite materials that exhibit excellent thermal stability while maintaining electrochemical performance. In general, adding fillers with desirable properties to the PVDF matrix offers an important improvement strategy [20,21,22,23].

Boron nitride (BN) has high thermal stability, a layered structure, and good thermal conductivity, effectively blocking the transfer of heat and oxygen. Phytic acid (PA) contains polyphosphate groups and abundant P elements, making it a typical P-based flame retardant that promotes char formation. Chitosan (CS) is a natural polysaccharide containing amino and hydroxyl groups, which can form hydrogen bond networks with PA to enhance compatibility while providing a carbon source for char formation [24,25,26]. The flame-retardant properties of these materials have been extensively studied in the context of their applications in suppressing or delaying combustion, and research efforts have been dedicated to investigating their performance mechanisms and compatibility with various polymeric matrices. For instance, the incorporation of CS-coated PA amide salt into PLA, a prevalent biodegradable polymer frequently constrained by its high flammability, effectively enhances PLA’s flame-retardant properties. Similarly, ammonium polyphosphate (APP)-CS/boron nitride (BN) composites, when employed as surface materials for substrates such as epoxy resins or polyurethane foams, demonstrate remarkable flame-retardant properties due to the synergistic effects between their components. Meanwhile, Zhang and co-workers crosslinked chitosan with bis-(4-formylphenyl)-phenyl-phosphonate; the resulting product was combined with ammonium polyphosphate and an organo-modified montmorillonite and incorporated into a thermoplastic polyurethane by melt mixing. It effectively addresses key fire safety challenges such as reducing heat release and inhibiting smoke production [27,28,29,30,31].

This study aims to develop functionalized BN (BN@PA-CS) through pre-treatment of BN, followed by layer-by-layer coating with PA and CS. The modified BN is then compounded with PVDF to regulate its thermal decomposition behavior, char layer structure, and combustion release characteristics, thereby achieving the goal of improving its flame-retardant characteristic [32,33,34,35,36,37].

## 2. Materials and Methods

### 2.1. Materials

Polyvinylidene fluoride (PVDF), hexagonal BN, PA (70% in H_2_O), and CS (deacetylation: >95%, *M_Ƞ_*: 55 × 104) were obtained from Aladdin Regents Co., Ltd., Shanghai, China. Concentrated HNO_3_ (AR), glacial acetic acid (CH_3_COOH ≥ 99.8%), and *N*,*N*-dimethylformamide (DMF, AR) were obtained from Sinopharm Chemical Reagent, Shanghai, China.

### 2.2. Preparation of BN@PA-CS and PVDF/BN@PA-CS Composite Materials

**Figure 1:**
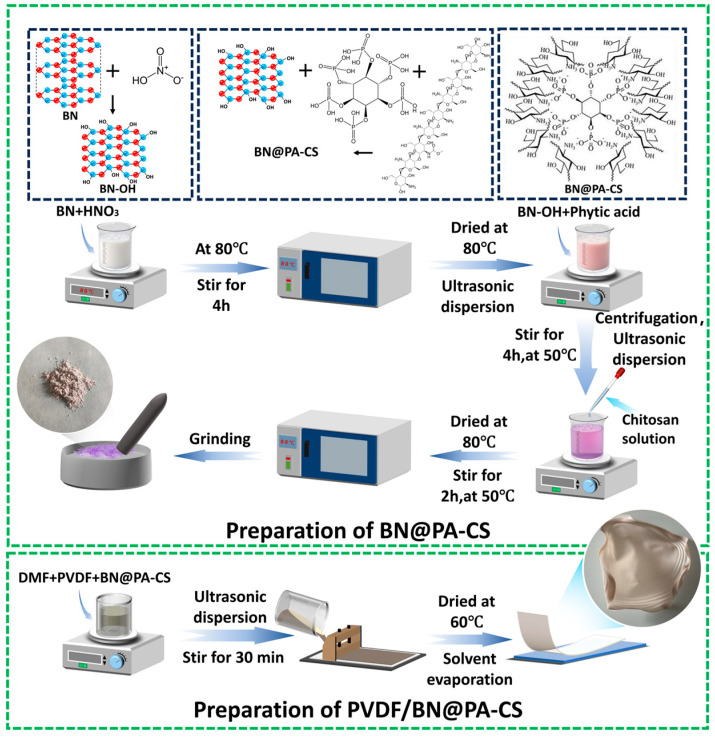
Schematic of the synthesis of BN@PA-CS and PVDF/BN@PA-CS.

The preparation of BN@PA-CS first involved the adsorption of BN using hydrogen bonding interactions between phytate and BN, followed by the assembly of a shell through electrostatic interactions between phytate (PA) and CS. The prepared BN@PA-CS was incorporated into PVDF to form a composite material. The specific experimental procedure is illustrated in Figure 1. First, 2 g of BN was mixed with concentrated HNO_3_ and stirred at 300 rpm and 80 °C for 4 h to acid-wash and oxidize the BN surface. Subsequent filtration and vacuum-drying at 80 °C for 12 h gave BN-OH. The BN-OH (1 g) was dispersed in 200 mL of deionized water using ultrasonication for 30 min, following which PA solution (75 mL of a 10 mg/mL) was added and the mixture was allowed to react for 4 h. After the reaction, the mixture was centrifuged and washed twice with deionized water and then redispersed in 200 mL of deionized water and ultrasonicated for 30 min. To this, 50 mL of a 10 mg/mL CS solution (dissolved in an aqueous acetic acid solution at a volume ratio of 22%) was added, and the mixture was stirred for 2 h. After the reaction, the mixture was centrifuged, and the solid was washed twice with deionized water. Then, this was placed in an 80 °C-oven and vacuum dried for 12 h. Following this, the solid was ground into powder to obtain BN@PA-CS [29]. PVDF (0.8 g), 15 mL of DMF, and modified BN at concentrations of 0, 5, 10, 15, and 20 wt% were added to a beaker. After ultrasonication for 15 min, the mixture was stirred at a constant temperature for 2 h. Once the solute was uniformly dispersed in the solvent, the mixture was transferred to a polytetrafluoroethylene plate and allowed to stand at room temperature for 12 h until the DMF solvent evaporated. The material was then placed in a constant-temperature forced-air oven at 60 °C to dry, and the resulting product PVDF/BN@PA-CS was removed using tweezers. The product was labeled as PVDF/BN@PA-CS *x* (*x* = 5, 10, 15, 20), where *x* indicates the amount of added BN (5, 10, 15, and 20 wt%).

### 2.3. Characterization

X-ray powder diffractometry (XRD, D/MAX2500, Rigaku Corporation, Tokyo, Japan) measurements were conducted using Cu Kα radiation (λ = 0.15418 nm) over 10–80° at a scan rate of 20°/min to investigate the structure of BN and BN@PA-CS.

To observe the morphologies and compositions of the BN@PA-CS, PVDF/BN@PA-CS, and char residues, field-emission scanning electron microscopy (SEM, FEI Quanta 250 FEG, FEI Inc., Valley City, ND, USA; recorded under high vacuum at a voltage of 20 kV) and transmission electron microscopy (TEM, FEI Tecnai G2 20, FEI Inc., Valley City, ND, USA) were performed.

Fourier-transform infrared (FTIR, Nicolet IS50, Thermo Fisher Scientific Inc., Waltham, MA, USA) spectroscopy was conducted from 400 to 4000 cm^−1^ at a resolution of 4 cm^−1^, with 32 scans per spectrum, to characterize the structures of BN and BN@PA-CS.

Thermogravimetric analysis (TGA, TG 219 F3, Netzsch Instruments Co. Ltd., Selb, Germany) was conducted at a constant scan rate of 10 °C/min under nitrogen at temperatures of 50–800 °C to obtain the TGA and differential thermogravimetry (DTG) thermograms. Each sample grade was subjected to three parallel measurements.

Differential scanning calorimetry (DSC, DSC 21 Netzsch Instruments Co. Ltd., Selb, Germany) was conducted at a heating rate of 10 °C/min to study the crystallization from the second heating curves of the PVDF composite films. Each sample grade was subjected to three parallel, precise measurements, and the final data were calculated as the average value of the three tests. The degree of crystallinity of the samples was determined using the formula Xc (%) = (∆Hm − ∆Hc) × 100/∆H^0^m, where Xc is the degree of crystallinity, ∆Hm is the enthalpy during melting, ∆Hc is the exothermic peak enthalpy, and ∆H^0^m is the enthalpy for the normalized enthalpy values (J/g) for 100% crystalline PVDF polymer, which is 219.7 J/g as reported in the literature [38].

An electromechanical tensile tester (Model 5900, Instron Corporation, Norwood, MA, USA) was employed to study the tensile performance of the PVDF with a constant rate of 50 mm/min; the length, width, and thickness of the samples were 33 mm, 6 mm, and 50–80 μm, respectively, and the thickness was measured using a thickness gauge.

The samples were analyzed using energy-dispersive X-ray spectroscopy (EDS, INCA Energy 350, Oxford Instruments, Abingdon, UK) at 20 kV.

The gases released during TGA were investigated using a Nicolet iS50 FTIR spectrometer. TGA-FTIR analysis was conducted using TGA coupled with FTIR spectroscopy. The FTIR spectrum of each sample was collected over 16 scans in the wavenumber range 500–4000 cm^−1^.

Microscale combustion calorimetry (DEATAK MCC-3, Fire Testing Technology Ltd., East Grinstead, UK) was conducted at temperatures ranging from room temperature to 900 °C at a heating rate of 1 °C/s to evaluate the flammability of the PVDF composite films.

Laser Raman spectroscopy measurements were performed at room temperature using a Renishaw RM2000 Raman apparatus. Samples were excited with a 514 nm laser, and spectra were recorded over a wavelength range of 200–4000 cm^−1^.

## 3. Results and Discussion

### 3.1. Structure and Morphology of BN@PA-CS

**Figure 2:**
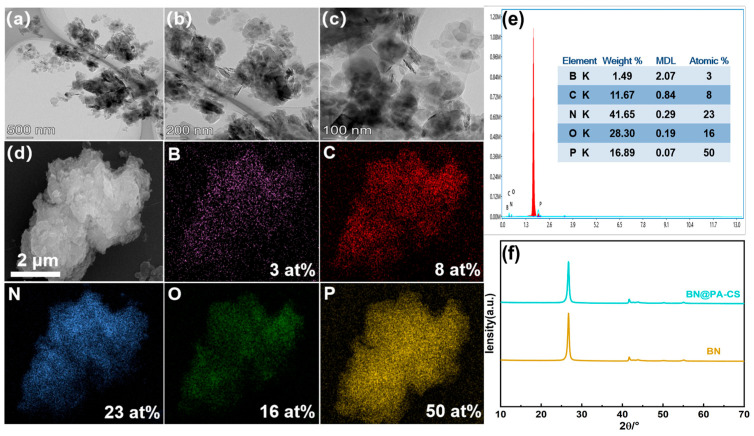
(**a**–**c**) TEM images and (**d**) SEM and EDS mapping of BN@PA-CS. (**e**) Quantitative EDS analysis table. (**f**) XRD spectra of BN and BN@PA-CS.

The TEM images shown in Figure 2a–c reveal dark nanoscale agglomerations on the BN surface, indicating that the surface was modified by PA-CS. The SEM image in Figure 2d shows wrinkles on the intact BN structure at the micrometer scale. A uniform distribution of B, C, N, P, and O is observed in the EDS mapping, corresponding to the elements in BN and PA-CS. Quantitative EDS analysis (Figure 2e) reveals a P content of 16.89%, which also confirms that the BN surface has a layer of PA-CS. The XRD peak positions of BN and BN@PA-CS (Figure 2f) largely overlap. This indicates that, while PA-CS forms a coating layer on the BN surface, it does not disrupt the crystalline phase of BN [31,32,33].

**Figure 3:**
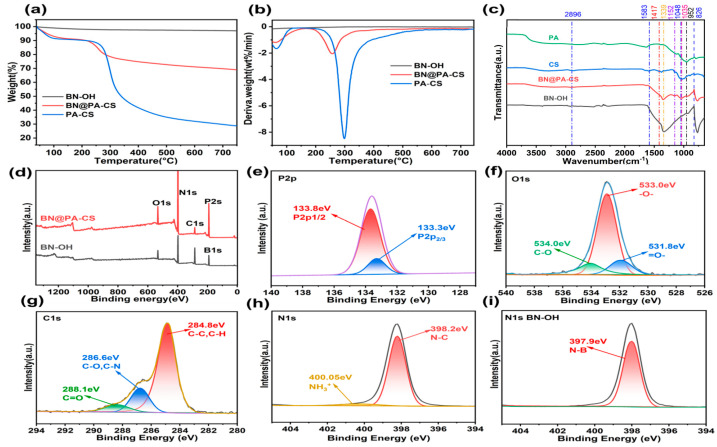
(**a**) TGA, (**b**) DTG curves, (**c**) FTIR, and (**d**) XPS spectra of BN and BN@PA-CS. (**e**–**h**) High-resolution XPS spectra of BN@PA-CS. (**i**) High-resolution XPS spectra of BN-OH.

As can be seen from Figure 3a and Table 1, BN-OH retains the high thermal stability of BN with no significant change in quality. The initial decomposition temperature (T5%) of BN@PA-CS is 75.3 °C, higher than that of PA-CS (66.3 °C). However, the maximum decomposition rate temperature (Tmax) is 257.5 °C, lower than that of PA-CS (298.7 °C). Furthermore, the residual mass of BN@PA-CS (69.1%) is considerably higher than that of PA-CS (28.8%). This suggests that BN prevents the PA-CS coating layer from decomposing too quickly at low temperatures and promotes the carbonisation of PA-CS at high temperatures. Additionally, the DTG curves in Figure 3b show that the thermal decomposition rate peaks of BN@PA-CS and PA-CS have a similar shape. Furthermore, FTIR analysis (Figure 3c) shows that the PA spectrum exhibits characteristic peaks of phosphate groups at approximately 1339 cm^−1^ and characteristic P-O absorption peaks at approximately 1048 cm^−1^. In the CS spectrum, the peaks at 2896, 1583, and 1417 cm^−1^ originate from the sugar ring structure and amino groups in CS. In the BN-OH spectrum, in addition to the characteristic peaks of the BN framework at approximately 1400 and 800 cm^−1^, a peak characteristic of the B-O bond appears at approximately 952 cm^−1^, indicating successful hydroxylation. Analysis of the BN, PA, and CS spectra revealed that the BN@PA-CS spectrum retained the characteristic peaks of the BN framework while exhibiting peaks characteristic of the phosphate, sugar rings, and amino groups in PA and CS between 1000 and 1500 cm^−1^. The X-ray photoelectron spectroscopy (XPS) profiles in Figure 3d–i show that BN@PA-CS not only exhibits peaks identical to those of BN, but also exhibits enhanced N 1s peaks owing to the PA-CS composite and enhanced P 2s peaks and B 1s overlap because of the introduction of P elements. High-resolution XPS spectra were obtained after peak fitting. The P 2p high-resolution spectrum shows two Spin–Orbit split peaks, P 2p_1_/_2_ and P 2p_3_/_2_, indicating that P primarily exists as phosphate ester functional groups, consistent with the P-containing functional groups in PA-CS. The O 1s high-resolution spectrum exhibits oxygen functional groups in different chemical states, which is consistent with the P-O and P=O groups in PA and the O-H group in CS. The high-resolution C 1s spectrum shows carbon in various chemical environments such as C–C, C–H, C–O, C–N, and C=O. These are all consistent with the chemical environment of carbon in PA-CS. The C=O peak may also originate from residual acetyl groups in the CS compound. The N 1s high-resolution spectrum shows that, compared to BN-OH, which only exhibits B-N peaks, BN@PA-CS additionally exhibits peaks corresponding to -NH_3_^+^ and C-N from PA-CS. Taken together, the XPS analyses confirm the successful preparation of the BN@PA-CS composite material [34,35,36].

### 3.2. Structure and Thermal Performance of PVDF/BN@PA-CS

**Figure 4:**
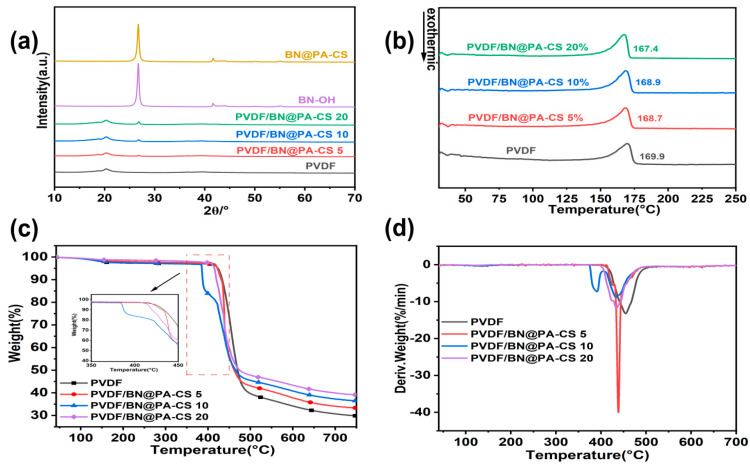
(**a**) XRD patterns of PVDF, PVDF/BN@PA-CS, and BN-OH. (**b**) DSC curves of PVDF and PVDF/BN@PA-CS. (**c**) TGA curves of PVDF and PVDF/BN@PA-CS. (**d**) DTG curves of PVDF and PVDF/BN@PA-CS.

As demonstrated in Figure 4a, the analysis indicates that pure polyvinylidene fluoride (PVDF) exhibits a characteristic peak at the diffraction angle of 20.23°, which is indicative of its crystal structure. Following the introduction of the BN@PA-CS composite filler, the XRD spectrum retained the 20.23° peak of PVDF, and concomitantly, new peaks of PA at 26.7° and BN at 41.6° were detected, with no interference from impurity peaks, thus confirming the successful preparation of the PVDF/BN@PA-CS composite material. As illustrated in Figure 4b–d, BN@PA-CS exerts a controllable influence on the thermal stability of PVDF. During the initial stage of thermal decomposition, the thermal weight loss curves of pure PVDF and the composite material exhibit a similar trend, indicating that the early thermal stability remains largely uncompromised. When combined with the data presented in Table 2, the T_5%_ of pure PVDF is determined to be 421.5 °C, with a T_max_ of 454.6 °C. For PVDF/BN@PA-CS 20, the T5% drops to 414.5 °C, and T_max_ drops to 434.5 °C, both showing a slight decrease; while the residual carbon content increases from 29.86% to 39.1%, with a relative increase of 30.94%, indicating that BN@PA-CS can promote the formation and stability of the carbonaceous layer at high temperatures. It is worth noting that Figure 4c and Table 2 clearly show that the initial decomposition temperature (T) of PVDF/BN@PA-CS10 is 385.5 °C, which is lower than that of pure PVDF (421.5 °C). A distinct characteristic peak also appears in Figure 4c. However, the decomposition rate of PVDF/BN@PA-CS5 is significantly faster, with an absolute value of 40.04. This indicates that insufficient additives result in a relatively weak interaction between the components. This results in two effects: small-molecular-weight substances decompose either earlier at low temperatures or rapidly at high temperatures. As illustrated in Figure 4b, with an increase in the content of BN@PA-CS, the melting point of the composite material decreases from 169.86 °C for pure PVDF to 167.44 °C, exhibiting a slight decrease without any discernible effect on the processing performance. As illustrated in Table 3, the crystallinity of pure PVDF and composite materials with varying filler contents stabilizes at approximately 37%, with a standard deviation of less than 1%. This finding suggests that BN@PA-CS is uniformly dispersed, without compromising the crystallization capability of PVDF, thereby ensuring the reliability of the material’s fundamental performance characteristics. In summary, an appropriate BN@PA-CS content (10–20 wt%) can promote PVDF crystallization and significantly enhance high-temperature thermal stability by reducing the decomposition rate and promoting char formation [37,38,39].

### 3.3. Morphology and Mechanical Property Analysis

**Figure 5:**
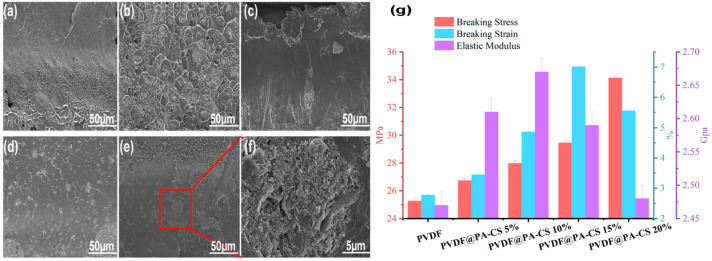
Cross-sectional SEM images of (**a**) PVDF, (**b**) PVDF/BN@PA-CS 5, (**c**) PVDF/BN@PA-CS 10, (**d**) PVDF/BN@PA-CS 15, and (**e**) PVDF/BN@PA-CS 20. (**f**) Magnified SEM image of the area marked with a red box in (**e**). (**g**) Columnar diagram of the mechanical properties of PVDF and PVDF/BN@PA-CS.

Figure 5 and Table 4 present the cross-sectional morphology and mechanical properties of PVDF and its composite films, respectively. Figure 5a shows the SEM image of the cross-section of pure PVDF, revealing a relatively smooth surface without any obvious structures indicating phase separation. However, the presence of internal stress results in microcracks, indicating non-uniformity of the cross-section. Figure 5b shows the SEM image of the cross-section of PVDF/BN@PA-CS 5 after the addition of BN@PA-CS. Phase separation is clearly visible here, with BN@PA-CS particles dispersed in the PVDF matrix forming a distinct interface structure. As the BN@PA-CS content increases, the phase separation phenomenon within the PVDF composite films weakens at additions of 10 and 15 wt% (Figure 5c,d), with BN@PA-CS dispersing more effectively in the PVDF matrix to form a more uniform composite structure. However, when the BN@PA-CS content reaches 15%, microcracks and particles begin to appear in the cross-section. Therefore, when the BN@PA-CS content reaches 20%, clear phase separation and particle aggregation occur (Figure 5e,f). Although the central part of the image retains a certain degree of uniform interface structure (Figure 5f), agglomerated structures are also evident. Figure 5g shows the macroscopic mechanical properties based on the above microstructural analysis. When the BN@PA-CS content is low, the BN@PA-CS particles disperse in the PVDF matrix to form a certain interface structure and play an interface-enhancing role. This increases the elastic modulus of the material and partially inhibits crack propagation, thereby improving both fracture stress and fracture strain. However, when the BN@PA-CS content increases further to 20 wt%, significant particle agglomeration or phase separation occurs, as evident from the SEM images. This agglomeration, caused by overfilling, leads to an increase in interface defects and a slight decrease in fracture stress. Nevertheless, the presence of a significant number of interface-enhancing phases in the matrix means that the fracture strain and fracture stress remain relatively high compared to pure PVDF [39,40].

### 3.4. Flame Retardancy of PVDF Composite Films and SEM Images of Residual Carbon

**Figure 6:**
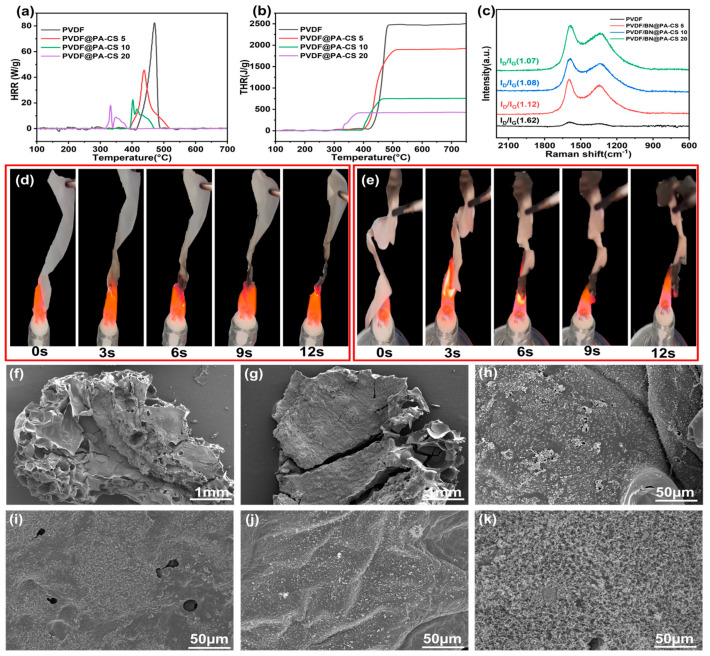
(**a**) HRR and (**b**) THR curves and (**c**) Raman spectra of PVDF and PVDF composite films. Combustion diagram of (**d**) pure PVDF and (**e**) PVDF/BN@PA-CS 20. (**f**–**j**) SEM images of residual carbon of PVDF, PVDF/BN@PA-CS 5, PVDF/BN@PA-CS 10, PVDF/BN@PA-CS 15, and PVDF/BN@PA-CS 20. (**k**) Enlarged SEM image of residual carbon of PVDF/BN@PA-CS 20.

To evaluate the flame retardancy of the composite films, we also conducted limiting oxygen index (LOI) and UL-94 tests, and present the results in Table 5. The LOI value represents the minimum oxygen volume percentage that can support material combustion, and serves as an indicator of flammability. The LOI of the PVDF composite film is higher than that of pure PVDF when BN@PA-CS is added. When ABN@PA-CS reaches 20 wt%, the LOI of the PVDF composite film reaches 38.3%. Furthermore, the UL-94 rating of pure PVDF film is NR. When the amount of BN@PA-CS added is more than 10%, the UL-94 rating becomes V0. Figure 6a shows the heat release rate (HRR) curves for PVDF and its composite films. As can be seen from the curves, the HRR of the PVDF/BN@PA-CS composite materials decreases with increasing BN@PA-CS addition. Additionally, the heat release curve of pure PVDF exhibits a high, sharp peak with a peak value of approximately 80 W/g. This indicates that pure PVDF decomposes rapidly after reaching 400 °C and exhibits poor flame-retardant properties. When BN@PA-CS is added, although the PA-CS decomposes prematurely, causing the peak position to shift forward and multiple peaks to appear, PA-CS acts as a carbon source. This promotes premature thermal decomposition and carbonization, covering the PVDF surface and reducing the thermal decomposition and oxidation of small molecules. The carbon layer formed by premature decomposition also serves as a barrier, delaying heat transfer. This is evident in Figure 6b, which shows that the total heat release (THR) of the PVDF composite films decreases significantly in a stepwise manner with the addition of different amounts of BN@PA-CS. This indicates that the barrier effect of the carbon layer effectively blocks heat transfer. Furthermore, the Raman spectrum in Figure 6c shows that pure PVDF has a high I_D_/I_G_ ratio and no distinct D or G peaks. However, with the addition of BN@PA-CS, distinct D and G peaks appear, and the I_D_/I_G_ ratio decreases significantly. This confirms the formation of the carbon layer and is consistent with previous results. The combustion diagrams of pure PVDF and PVDF/BN@PA-CS 20 in Figure 6d,e show that the carbon layer formed is thicker (at the macro level) when the BN@PA-CS content is 20 wt%. Combined with the residual carbon SEM images in Figure 6f–k, it can be seen that the residual carbon of pure PVDF in Figure 6f is loose and porous with thin edges. Figure 6g shows that after adding BN@PA-CS, the carbon layer becomes denser and more compact with fewer thin edges. However, upon closer inspection at a smaller scale (Figure 6h), the surface of the carbon layer appears relatively loose and contains numerous small pores. As the amount of BN@PA-CS increases, the carbon layer gradually thickens and becomes more compact, with the pores gradually decreasing in size and eventually disappearing. However, it is worth noting that in Figure 6k, the carbon layer of PVDF/BN@PA-CS 20 begins to loosen. This is due to the deteriorating compatibility between BN@PA-CS and PVDF at high concentrations, which leads to a loosening of the carbon layer structure and an increase in defects. This explains why the peak values of HRR and THR, as well as the I_D_/I_G_ ratio in the Raman spectrum, are similar for PVDF/BN@PA-CS 20 and PVDF/BN@PA-CS 10 [41,42].

### 3.5. Condensed Phase Analysis

**Figure 7:**
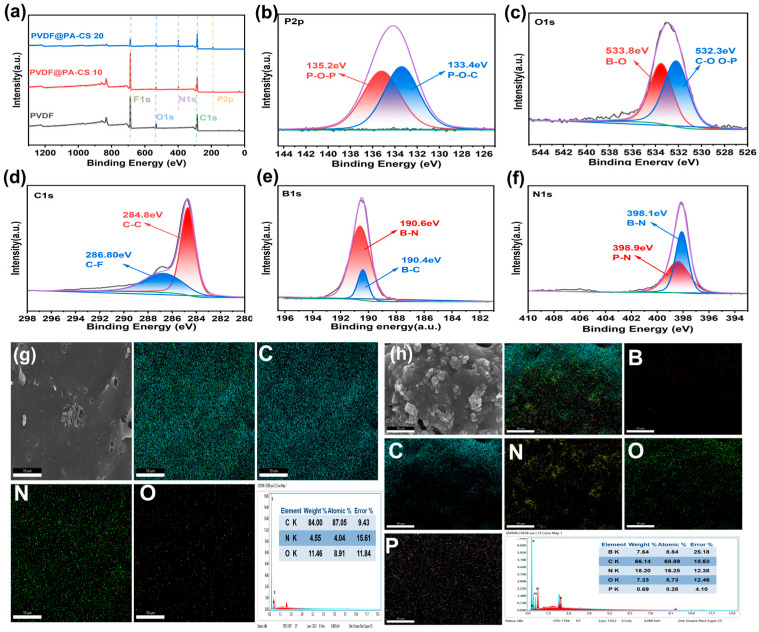
(**a**) XPS full spectrum of the char layer after combustion for PVDF, PVDF/BN@PA-CS 10, and PVDF/BN@PA-CS 20. (**b**–**f**) XPS high-resolution spectra of PVDF/BN@PA-CS 20. (**g**) SEM image and EDS mapping of PVDF. (**h**) SEM image and EDS mapping of PVDF/BN@PA-CS 15.

Figure 7 shows the EDS mapping and XPS spectra of PVDF and its composite residues. The XPS full spectrum (Figure 7a) shows that the peaks representing elements such as N and P begin to appear or intensify with increasing BN@PA-CS content. This indicates the presence of P- and N-containing compounds in the residual carbon of PVDF/BN@PA-CS 20. The high-resolution XPS spectra of PVDF/BN@PA-CS 20 shown in Figure 7b–f are diverse. The F 1s high-resolution XPS spectrum in Figure 7d shows a single C-F chemical state. This indicates that the main combustion products in the residual carbon are primarily PVDF. The P 2p high-resolution spectrum in Figure 7b shows P-O-P and P-O-C chemical states, indicating that substances similar to phosphates formed by the thermal decomposition of PA are present in the residual carbon. The B 1s high-resolution spectrum in Figure 7e shows B-N and B-C chemical states, indicating that BN is present in the residual carbon. This is consistent with the high thermal stability of BN. Figure 7f shows the high-resolution N 1s spectrum, which, in addition to the previously mentioned B–N chemical species, also exhibits N–H chemical species. This indicates the formation of ammonium salts in the residual carbon. Figure 7h,i show the EDS profiles of the pure PVDF carbon layer and the PVDF/BN@PA-CS 15 carbon layer, respectively. Compared to the pure PVDF carbon layer, the PVDF/BN@PA-CS 15 carbon layer shows the addition of P elements and increased N content. These results are consistent with the XPS analysis and further confirm the presence of P- and N-containing compounds in the residual carbon [27,43,44].

### 3.6. Gas Phase Analysis

**Figure 8:**
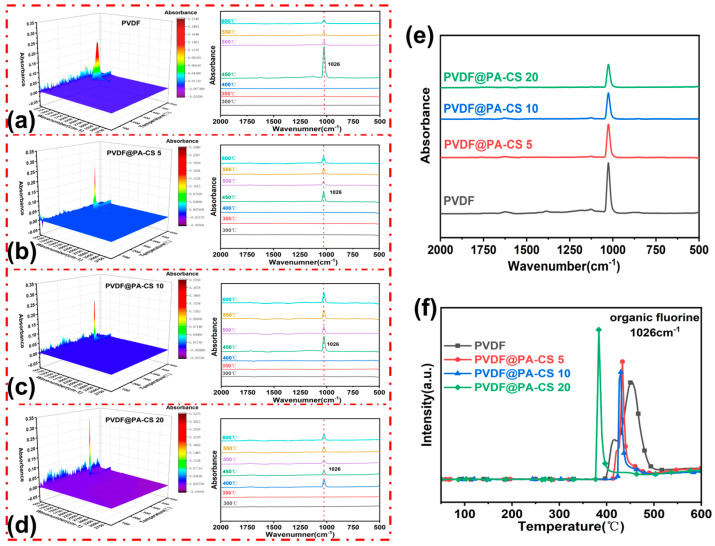
(**a**–**d**) Three-dimensional TG-FTIR spectra of PVDF and PVDF/BN@PA-CS. (**e**) Release curve at the maximum thermal decomposition temperature with PVDF and PVDF/BN@PA-CS. (**f**) Temperature-dependent curve of organic fluorine thermal decomposition products.

Figure 8 shows the three TG-FTIR test average results for PVDF and its composite films. As shown in Figure 8a–d, the characteristic absorption peaks of PVDF and its composite materials are concentrated at 1026 cm^−1^, which corresponds to the stretching vibration of C–F bonds. Pure PVDF reaches its peak absorption at approximately 450 °C; however, the absorbance decreases rapidly when the temperature exceeds this value. This phenomenon is not observed in the PVDF/BN@PA-CS composite material, indicating that pure PVDF decomposes rapidly above a certain temperature. When BN@PA-CS is added, PA-CS forms a carbon layer that delays the decomposition of PVDF and suppresses the volatilization of fluorinated compounds. Combining the release curves at the maximum decomposition temperature (Figure 8e) shows that the peak of the C-F bond decreases significantly with the addition of an increased amount of BN@PA-CS. Figure 8f shows the temperature-dependent decomposition curve of organic fluorine compounds. After adding BN@PA-CS, the curve shifts toward lower temperatures and the peak width narrows. As the added amount increases, the shift becomes more pronounced and the peak width gradually narrows. This indicates that PA-CS decomposes before reaching the PVDF decomposition temperature, and that its decomposition inhibits PVDF decomposition. This result is consistent with previous analyses [45,46].

### 3.7. Proposed Flame-Retardant Mechanism

**Figure 9:**
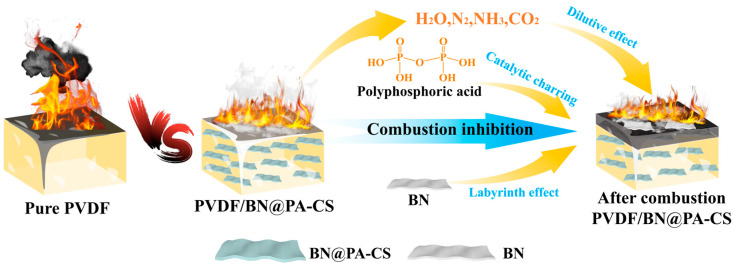
Schematic of the fire-retardant mechanism of BN@PA-CS.

Previous analyses suggest that BN@PA-CS exhibits effective flame-retardant properties when incorporated into PVDF (Figure 9). First, the layered structure of BN disperses within the PVDF matrix, creating a ‘maze’ effect that extends the heat diffusion path and hinders flame propagation. Second, the phosphate groups in PA catalyze the carbonization of PVDF during combustion, forming a dense carbon layer that isolates oxygen and heat. Overall, introducing BN@PA-CS enables a range of synergistic effects through multiple mechanisms during PVDF combustion, thereby enhancing the fire safety of the PVDF/BN@PA-CS composite material [47].

## 4. Conclusions

In this study, multifunctional composite particles (BN@PA-CS) with excellent flame retardancy and interfacial compatibility were prepared by coating BN with PA and CS. These BN@PA-CS particles were then incorporated into PVDF to enhance its flame-retardant and mechanical properties. The mechanical properties indicate that uniform dispersion of the BN@PA-CS particles enhances the tensile strain and elastic modulus of the PVDF matrix. Microcalorimetric tests demonstrate that the flame-retardant performance of the PVDF/BN@PA-CS composite materials is significantly enhanced. According to TG-FTIR results, the addition of BN@PA-CS suppresses the generation of volatile substances containing toxins. During combustion, the catalytic carbonization, maze, and dilution effects of BN@PA-CS reduce the fire hazard of the PVDF/BN@PA-CS composite materials. In conclusion, this study provides new insights into the green synthesis of bio-based nanoparticles and their application in PVDF.

## Figures and Tables

**Table 1:** TGA results for BN, BN@PA-CS and PA-CS.

Sample	T5% (°C)	Tmax (°C)	Residual Mass (%)
BN	/	/	97.2
BN@PA-CS	75.3 ± 0.6	257.5 ± 0.4	69.1
PA-CS	66.3 ± 0.5	298.7 ± 0.5	28.8

**Table 2:** TGA results for PVDF and PVDF/BN@PA-CS.

Sample	T_5%_ (°C)	T_max_ (°C)	Rate of T_max_ (wt.% min^−1^)	Yc (wt%)
PVDF	421.5 ± 0.35	454.6 ± 0.25	−13.24 ± 0.50	29.86 ± 0.50
PVDF/BN@PA-CS 5	421.8 ± 0.45	438.5 ± 0.55	−40.04 ± 0.50	33.34 ± 0.35
PVDF/BN@PA-CS 10	385.5 ± 0.50	432.2 ± 0.50	−8.72 ± 0.55	36.49 ± 0.45
PVDF/BN@PA-CS 20	414.5 ± 0.50	434.5 ± 0.45	−11.75 ± 0.45	39.10 ± 0.55

**Table 3:** DSC results for PVDF and PVDF/BN@PA-CS.

Sample	Melting Point/°C	Melting Enthalpy/(J·g^−1^)	Crystallinity (%)
PVDF	169.86 ± 0.50	34.89 ± 0.54	37.1 ± 0.65
PVDF/BN@PA-CS 5	168.71 ± 0.54	32.00 ± 0.55	34.0 ± 0.55
PVDF/BN@PA-CS 10	168.93 ± 0.55	34.97 ± 0.58	37.2 ± 0.55
PVDF/BN@PA-CS 20	167.44 ± 0.54	35.14 ± 0.45	37.4 ± 0.58

**Table 4:** Mechanical properties of PVDF and PVDF/BN@PA-CS.

Samples	Breaking Stress (MPa)	Breaking Strain (%)	Elastic Modulus (GPa)
PVDF	25.27 ± 0.65	2.78 ± 0.55	2.47 ± 0.45
PVDF/BN@PA-CS 5	26.74 ± 0.55	3.45 ± 0.65	2.61 ± 0.65
PVDF/BN@PA-CS 10	27.98 ± 0.45	4.86 ± 0.55	2.67 ± 0.65
PVDF/BN@PA-CS 15	29.45 ± 0.55	7.01 ± 0.55	2.59 ± 0.58
PVDF/BN@PA-CS 20	34.13 ± 0.56	5.56 ± 0.50	2.48 ± 0.57

**Table 5:** UL-94 and LOI Test Results for PVDF composite films.

Sample	PVDF Content (wt%)	BN@PA-CS Content (wt%)	UL-94 Rating	LOI (%)
PVDF	100	0	NR	32.6
PVDF/BN@PA-CS 5	95	5	V-2	34.1
PVDF/BN@PA-CS 10	90	10	V-0	36.8
PVDF/BN@PA-CS 15	85	15	V-0	37.2
PVDF/BN@PA-CS 20	80	20	V-0	38.3

## Data Availability

The data presented in this study are available on request from the corresponding author.
